# Inference of Historical Population-Size Changes with Allele-Frequency Data

**DOI:** 10.1534/g3.119.400854

**Published:** 2019-11-07

**Authors:** Michael Lynch, Bernhard Haubold, Peter Pfaffelhuber, Takahiro Maruki

**Affiliations:** * Biodesign Center for Mechanisms of Evolution, Arizona State University, Tempe, AZ 85287,; † Department of Evolutionary Genetics, Max Planck Institute for Evolutionary Biology, Plön 24306, Germany, and; ‡ Faculty of Mathematics and Physics, University of Freiburg, Freiburg 79104, Germany

**Keywords:** coalescent, *Daphnia*, demographic history, effective population size, site-frequency spectrum

## Abstract

With up to millions of nearly neutral polymorphisms now being routinely sampled in population-genomic surveys, it is possible to estimate the site-frequency spectrum of such sites with high precision. Each frequency class reflects a mixture of potentially unique demographic histories, which can be revealed using theory for the probability distributions of the starting and ending points of branch segments over all possible coalescence trees. Such distributions are completely independent of past population history, which only influences the segment lengths, providing the basis for estimating average population sizes separating tree-wide coalescence events. The history of population-size change experienced by a sample of polymorphisms can then be dissected in a model-flexible fashion, and extension of this theory allows estimation of the mean and full distribution of long-term effective population sizes and ages of alleles of specific frequencies. Here, we outline the basic theory underlying the conceptual approach, develop and test an efficient statistical procedure for parameter estimation, and apply this to multiple population-genomic datasets for the microcrustacean *Daphnia pulex*.

Because polymorphisms with different allele frequencies arise at different average times in the past, information on the amount of variation associated with different allele-frequency classes in a population sample can provide insight into the history of population-size change. This is especially true for neutral variants, whose temporal dynamics depend only on stochastic sampling effects. This simple idea has led to the development of several technical and computationally demanding approaches for estimating historical changes in the sizes of populations, either from patterns of segregating variation at the single-nucleotide level or from information on linkage disequilibrium between nucleotide sites (Strimmer and Pybus 2001; Hayes *et al.* 2003; Tenesa *et al.* 2007; Gutenkunst *et al.* 2009; Li and Durbin 2011; Bhaskar *et al.* 2015; Liu and Fu 2015; Gattepaille *et al.* 2016; Weissman and Hallatschek 2017). All of these methods make numerous assumptions, some of which can be difficult to validate (*e.g.*, the negligible influence of nonneutral sites), are almost certainly violated (*e.g.*, linearity of the relationship between the recombination rate and physical distance betwen sites), and/or require information that is not available for most species (*e.g.*, the identification of derived *vs.* ancestral alleles). Moreover, it remains to be seen whether simpler, more intuitive approaches might yield results that perform to a comparable (or even greater) degree of accuracy.

The approach taken here is conceptually straight-forward, the main biological assumptions being that the sites underlying the analysis have evolved in a neutral fashion for a considerable number of generations (roughly speaking, for at least four times the current effective population size, which is the expected coalescence time to common ancestry under current conditions), and that there be no substantial population structure. All aspects of the analysis are based on samples of the site-frequency spectrum (SFS) for such sites. Letting *n* be the number of sampled haploid genomes (typically twice the number of individuals in a sample from a diploid population), the number of polymorphic genomic sites with *r* copies of the derived allele is denoted $G_{r}$, where r=1 to (n−1). The number of monomorphic sites is $G_{0}$, and the SFS is defined as $G_{r}/G,$ where *G* is the total number of monomorphic and polymorphic sites evaluated across the genome. In the following, a mutation in class *r* will be referred to as *r*th order, with r=1 denoting singletons, r=2 doubletons, etc.

The methods that follow, which adhere to the theory utilized in the stairway method of Liu and Fu (2015), are based on three principles. First, the frequencies of sites residing within different classes are functions of the historical pattern of effective population size ($N_{e}$) – all other things being equal, increases in $N_{e}$ elevate the probability of an allele residing in a particular polymorphic state, but the relative frequency also depends on the sequence of $N_{e}$ experienced by all other allelic classes. Second, the SFS for neutral sites scales with the mutation rate per site per generation (*u*) (Kimura 1983), so if quantitative information on $N_{e}$ is desired, an estimate of *u* is required. Third, the frequency of a mutation provides information on its age – under the process of neutral drift, the time for a new mutation to reach a frequency class is a monotonic function of the frequency, although there is considerable noise around the expectation.

The goal here is to use these principles to determine the long-term series of effective population sizes most compatible with the SFS, and given the measures of interval-specific $N_{e}$ to estimate the temporal history of past population-size changes experienced by segregating polymorphisms. We present analytical solutions for a broad set of genealogical features of a sample that are independent of the demographic history, and use this theory to develop estimators for the average age of single-nucleotide polymorphisms within each frequency class and the average $N_{e}$ experienced during their history. These results are worked out for the case of the unfolded SFS, and extended to the folded SFS, which summarizes the incidence of the minor-allele frequency classes, as investigators only rarely know which allele segregating at a locus is derived. A computationally efficient method for estimating such parameters is presented, validated with comparisons to computer-simulated data, and applied to large population-genomic data sets of *Daphnia pulex*.

## Theory

The Kingman (1982) coalescent provides the theoretical basis for all that follows. Under this view, members of a genealogy of *n* samples (assumed to be $\ll N_{e}$) randomly coalesce each generation until the entire genealogy has congealed to one common ancestor at the base of the tree after the (n−1)th coalescence event. Although the number of possible tree topologies is enormous with large sample sizes, many of the summary features of the coalescent are known (Hein *et al.* 2005; Wakeley 2009).

**Figure 1:**
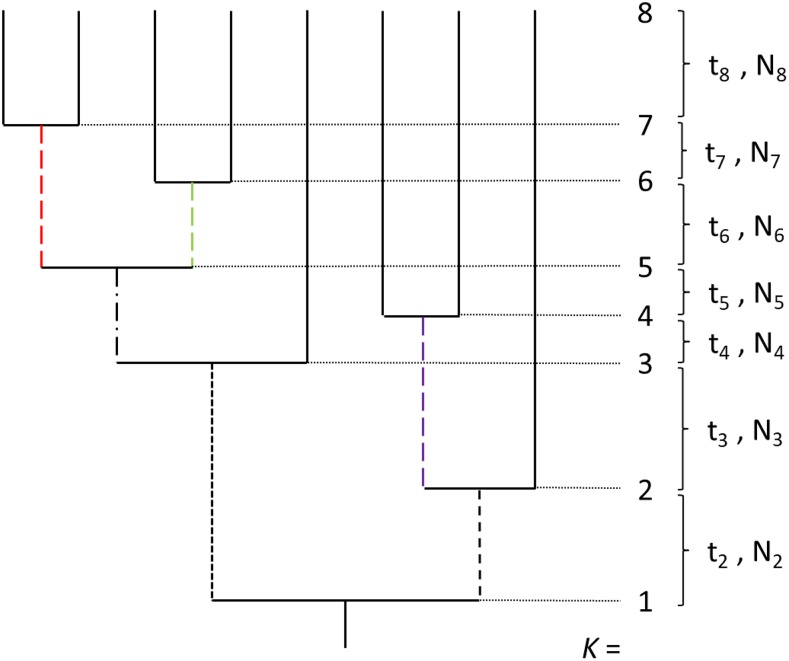
A genealogy comprising a sample size of n=8, with the *k* values denoting the ordered coalescence events (k=1 representing the root of the tree). The external branches, which can contain only singleton mutations, are given as solid black lines. There are three internal branches of order r=2 (upon which doubleton mutations must reside, denoted with large dashes); the red, green, and purple doubleton branches have starting and ending levels of (7,5), (6,5), and (4,2), respectively. The tree also contains single branches of orders r=3 (tripletons, medium dashes), 4 (quadrupletons, dot/dashes), and 5 (quintupletons, small dashes). The consecutive coalescence times are denoted by $t_{k}$, with $N_{k}$ denoting the effective population size from level *k* to k−1.

Here, we are concerned with the average features of alleles within different frequency classes (r=1 to n−1) in the sample, which requires an understanding of the nature of the branch segments on which mutations of the different classes can reside. These probabilistic features can be summarized with a knowledge of $P_{k,k-x}\left(r\right)$, the probability that an allele (SNP, or single nucleotide polymorphism) with frequency r/n resides on an uninterrupted branch starting at level *k* and ending at level (k−x), where k=n denotes the branch tips and k=1 denotes the base of the tree (Figure 1). For any class of mutations, the underlying branch segments can start as early as level (n−r+1) (singleton branches always start at level *n*) and can end as deeply as level 1. This means that internal branches starting at level *k* can span up to k−1 possible coalescence events in the tree. Each coalescence event can potentially be associated with a unique effective population size.

A key point is that the $P_{k,k-x}\left(r\right)$ coefficients are completely independent of the underlying demographic history, as the coordinates are simply denoted by the enumerated coalescence events, and are functions of only the sample size (*n*) and the allele class (*r*). Only the branch lengths are functions of the population size. As described below, the full set of coefficients (derived in the Appendix) provide the basis for analytical expressions for various useful statistical features of SNPs. Here, we adhere to the infinite-sites model, so that each new mutation arising on a genealogy is assumed to appear at a unique site, with the mutation rate to a novel SNP being equal to *u* per site per generation.

### An interval-specific view of $N_{e}$

We start with the assumption that *n* sequences have been sampled randomly (from n/2 diploid individuals, or *n* haploids) at each of *L* nucleotide sites known to behave in an effectively neutral manner. Under neutrality, for a population with constant effective size, the expected number of sites occupying frequency class *r* in an unfolded site-frequency spectrum is

$$E\left[G_{r}\right]=\frac{\theta \cdot L}{r}$$ (1)

where $\theta =4N_{e}u$ (for diploids, assumed here, or $2N_{e}u$ for haploids), with *u* being the mutation rate per nucleotide site per generation (Watterson 1975; Fu 1995; Ewens 2004; Messer 2009). Here, we take a more refined approach by explicitly evaluating the way in which each SNP class reflects the historical series of coalescence events within a sample, averaging over all possible coalescent topologies for a sample of size *n*. Looking into the past, a tree composed of *n* sequences (branch tips) experiences (n−1) coalescence events to the base, and to each level *k* we assign an effective population size $N_{k}$, such that $N_{n}$ denotes the average effective size between the branch tips and the first coalescence event, $N_{n-1}$ represents the average in the interval between the first and second coalescence events, and so on. At level *k*, the expected time (in generations) to the next coalescence event into the past is

$$t_{k}=\frac{4N_{k}}{k\left(k-1\right)}$$ (2)

generations. For example, starting with k=n, $t_{n}=4N_{n}/\left[n\left(n-1\right)\right]$ denotes the expected number of generations until the first coalescence event in the sample; and the second coalescence event, which depends on $N_{n-1},$ is obtained by setting k=n−1, *i.e.*, $t_{n-1}=4N_{n-1}/\left[\left(n-1\right)\left(n-2\right)\right].$

Given knowledge of the expected internal features of the coalescent, for each SNP frequency class, the expected value of $G_{r}$ can be expressed as a function of the full set of relevant $N_{k},$ which determine the lengths of branches upon which mutations arise. This also requires expressions for the expected number of branches of order *r* at each relevant level of the coalescent, averaged over all possible random genealogies in a sample of size *n*. These are derived in the Appendix.

As an example, consider the simplest case of the singleton class. All singleton mutations must be present on external branches, which always start at level *n* but may end at any level in the genealogy from n−1 (the first coalescent) to 1 (extending to the base of the tree). The expected number of singletons in the sample is

$$E\left[G_{1}\right]=4unL\sum_{k=1}^{n-1}\frac{S_{n,\left(n+1-k\right)}\cdot N_{n+1-k}}{\left(n-k\right)\left(n+1-k\right)},$$ (3a)

where $S_{n,\left(n-x\right)}$ is the probability of a singleton branch (starting at level *n*) not having coalesced by level (n−x). This expression is equal to the sum of the product of the expected number of singleton branches surviving at each level and the length of the subsequent coalescence interval, all multiplied by the probability of a mutation arising per site per generation. Using the expression for $S_{n,\left(n-x\right)}$, Equation (A3) in the Appendix, the preceding expression simplifies to

$$E\left[G_{1}\right]=\frac{4uL}{\left(n-1\right)}\sum_{k=2}^{n}N_{k}=4uL\bar{N}\left(n\right),$$ (3b)

where $\bar{N}\left(n\right)$ is the arithmetic average of the interval-specific $N_{i}$ from the top ($N_{n}$) to the bottom ($N_{2}$) levels of the tree. This result applies regardless of the mode of population-size change, showing that an estimate of the arithmetic average $N_{e}$ across all coalescence events is provided by the incidence of singletons, *i.e.*, as $\bar{N}\left(n\right)=G_{1}/\left(4uL\right)$, where $G_{1}$ denotes an estimate of the number of singletons.

Things are more complicated for the higher-order site-frequency classes because internal branches no longer initiate at the same levels. However, by extension from Equation (3b), one can infer that the probability of a mutation arising on a single branch starting at level *k*, allowing for variable ending points, is $4u\bar{N}\left(k\right)/k,$ where $\bar{N}\left(k\right)$ is the arithmetic average of the interval-specific $N_{i}$ starting at level *k* and descending down to the base of the tree. From Equation (A7), the expected number of order-*r* branches starting at level *k* is $\left[\left(r-1\right)/\left(n-k\right)\right]\cdot W_{k}\left(r\right)$, where $W_{k}\left(r\right)$ is a coefficient defining the expected number of segments of order *r* present at level k, given by Equation (A5). Summing these contributions over all levels,

$$E\left[G_{r}\right]=4uL\left(r-1\right)\sum_{k=2}^{n-r+1}\frac{W_{k}\left(r\right)}{k\left(n-k\right)}\cdot \bar{N}\left(k\right),$$ (4a)

which can also be written as

$$\begin{array}{c} E\left[G_{r}\right]=\frac{4uL\cdot \left(n-r-1\right)!}{\left(n-1\right)!}\sum_{k=2}^{n-r+1}\frac{\left(n-k\right)!}{\left(n-k-r+1\right)!}\cdot N_{k}\\ \frac{=4uL}{r}\frac{1}{\left(\begin{array}{c} n-1\\ r \end{array}\right)}\sum_{k=2}^{n-r+1}\left(\begin{array}{c} n-k\\ r-1 \end{array}\right)N_{k}, \end{array}$$ (4b)

also obtained by Liu and Fu (2015). These expressions show that the expected frequencies of all mutation classes are defined by differentially weighted averages of the interval-specific $N_{e}$. When r=1, Equation (4b) yields (3b), and with constant $N_{e}$, it reduces to $E\left[G_{r}\right]=4N_{e}uL/r,$ in accordance with Equation (1); considerable simplification is also possible if many adjacent $N_{k}$ have the same values (see Supplemental Material).

Before proceeding, recall that there are two forms of a site-frequency spectrum. The unfolded distribution, described above, requires information on the ancestral allelic states of each SNP site, ideally inferred from at least two suitably distant outgroup species (Keightley and Jackson 2018). Such a distribution is a summary of all sites having derived-allele frequencies 1/n to (n−1)/n. If ancestral allelic states are unknown, as is often the case, one must work with the folded site-frequency spectrum, which summarizes the minor-allele frequencies in classes 1/n to 1/2. The folded site-frequency spectrum, with 1≤r≤n/2, is defined as

$$F_{r}=G_{r}+G_{n-r},$$ (5)

with $F_{n/2}=G_{n/2}$ if *n* is even.

### Average age of a SNP

Whereas the previous results are concerned with the demographic history of the population, an alternative viewpoint considers the average ages and demographic features of SNPs of various frequencies. Once the interval-specific estimates of $N_{k}$ are available, the statistical machinery developed in the Appendix can be used to infer both order-specific measures. There, we show that for an unfolded SFS the average age (in generations) of an *r*th-order SNP in terms of the historical effective population sizes is

$$E\left[A_{r}\right]=\frac{4\sum_{k=2}^{n}N_{k}\left(\begin{array}{c} n-k\\ r-1 \end{array}\right)\sum_{\ell =k}^{n}\frac{N_{\ell }}{\ell \left(\ell -1\right)}}{\sum_{k=2}^{n}N_{k}\left(\begin{array}{c} n-k\\ r-1 \end{array}\right)}.$$ (6a)

The expected second moment is expressed as

$$E[A_{r}^{2}]=\frac{32\sum_{k=2}^{n}N_{k}\left(\begin{array}{c} n-k\\ r-1 \end{array}\right)\sum_{\ell =k}^{n}\frac{N_{\ell }}{\ell \left(\ell -1\right)}\sum_{m=\ell }^{n}\frac{N_{m}}{m\left(m-1\right)}}{\sum_{k=2}^{n}N_{k}\left(\begin{array}{c} n-k\\ r-1 \end{array}\right)},$$ (6b)

so the variance of ages of SNPs can be obtained as $\text{Var}\left(A_{r}\right)=E\left[A_{r}^{2}\right]-E^{2}\left[A_{r}\right]$, after substituting in the estimates for the $N_{k}.$

Although the preceding expressions apply to an unfolded SFS, where the designated alleles are known to be derived (by use of appropriate outgroup species for identifying ancestral allelic states), studies without such a luxury must rely on a folded SFS. In this case, each frequency class will be a mixture of derived and ancestral alleles with different average ages. For low-frequency alleles in large samples, almost the entire set of sampled SNPs will consist of derived alleles, and the preceding expressions can still be used to obtain reasonably precise estimates. This follows from Equation (1), which shows that the expected frequency of SNPs of order *i* is inversely proportional to *i*. Thus, for i=r and n−r, the fractional contribution of the former to the folded distribution is of order (n−r)/n provided the $N_{e}$ associated with the two classes are not greatly different (and larger than this if $N_{e}$ is larger for the younger alleles). For r≪n, almost all of the SNPs within folded class *r* will be derived alleles.

A more precise approach is to explicitly treat the frequencies of the folded distribution as mixtures of classes of derived alleles of order *r* and ancestral alleles of order n−r, with respective relative probabilities $p_{d}$ and $p_{a}=1-p_{d}$. The expected age of a SNP of order *r* in a folded SFS can then be written as

$$E\left[A_{r}^{\text{*}}\right]=\left(p_{d}\cdot E\left[A_{r}\right]\right)+\left(p_{a}\cdot E\left[A_{n-r}\right]\right),$$ (7a)

where $p_{d}=E\left[G_{r}\right]/\left(E\left[G_{r}\right]+E\left[G_{n-r}\right]\right).$ The components of $p_{d}$ can be estimated by substitution of the estimates for the $N_{k}$ into Equation (4b), and $E\left[A_{r}\right]$ and $E\left[A_{n-r}\right]$, and their expected squared values, are estimated by use of Equations (6a,b). The variance of $A_{r}^{\text{*}}$ is then

$$\text{Var}\left(A_{r}^{\text{*}}\right)=\left[p_{d}^{2}\cdot \text{Var}\left(A_{r}\right)\right]+\left[p_{a}^{2}\cdot \text{Var}\left(A_{n-r}\right)\right].$$ (7b)

### Average $N_{e}$ of a SNP

For a population experiencing temporal changes in size, alleles of different order will generally experience different long-term effective population sizes from birth to the present. Letting the population size at time *s* in the past be N(s), the expected average population size experienced by an allele of frequency *r* is

$$E\left[P_{r}\right]=\frac{E\left[\int_{0}^{A_{r}}N\left(s\right)ds\right]}{E\left[A_{r}\right]}.$$

If one has information on the ancestral states of alleles, and hence an unfolded site-frequency spectrum, the mean $N_{e}$ experienced by an allele of order *r* can be obtained from the theoretical results on the mean time spent in different intervals. Weighting of the interval-specific durations by their associated $N_{e}$ values leads to

$$E\left[P_{r}\right]=\frac{\sum_{k=2}^{n}N_{k}\left(\begin{array}{c} n-k\\ r-1 \end{array}\right)\sum_{\ell =k\;}^{n}\frac{4N_{\ell }^{2}}{\ell \left(\ell -1\right)}}{\sum_{k=2}^{n}N_{k}\left(\begin{array}{c} n-k\\ r-1 \end{array}\right)\sum_{\ell =k\;}^{n}\frac{4N_{\ell }}{\ell \left(\ell -1\right)}}.$$ (8)

see Appendix. With a folded site-frequency spectrum, the weighting approach used for the age of an allele in the preceding section can be applied using the definitions of $p_{d}$ and $p_{a}$, as well as $E\left[P_{r}\right]$ and $E\left[P_{n-r}\right]$ as defined in Equation (8). Nonetheless, with large sample sizes, the proposed approach is still expected to yield fairly accurate information on the average $N_{e}$ of rare alleles. This again follows from Equation (1), which shows that the expected frequency of SNPs of order *i* is inversely proportional to *i*.

## Estimation procedure

The results summarized in Equation (4b) amount to a series of n−1 equations, each a function of the mutation rate, *u*, and one or more of the interval-specific effective population sizes, $N_{i}$. Thus, in principle, working backward, one could apply the elements of the observed SFS to Equation (4b) to recursively estimate the full set of $N_{i}$ necessary to account for the data, *i.e.*, solving a set of n−1 equations for n−1 unknowns. However, with large numbers of unknowns and imperfectly estimated $G_{r}$, such an approach leads to aberrant results, including negative population-size estimates. Moreover, in the case of a folded site-frequency spectrum, the number of possible unknown population sizes exceeds the number of observed frequency classes.

It then becomes necessary to pool adjacent population sizes so as to reduce the number of parameters to be estimated. Consistent with Liu and Fu (2015), we have adopted a stepwise procedure, implemented in a likelihood framework. Consider a sampled site-frequency spectrum given by $G_{1},\ldots ,G_{n-1}$, where $G_{1}$ is the number of singletons, $G_{2}$ the number of doubletons, etc. With *L* sampled sites, the number of monomorphic sites is $G_{0}=L-G_{1}-\cdots -G_{n-1}$. For any set of interval-specific population sizes, Equations (4b,5) give the expected frequencies of SNPs in the full set of classes. Using a composite-likelihood approach, *i.e.*, assuming that the elements of the sampled SFS are all essentially independent and Poisson distributed with parameters equal to the frequency expectations times *L*, the likelihood function is given in Supplemental Material.

We have implemented the above procedure in the program epos (Estimating POpulation Sizes), which runs under the UNIX command line. The C sources of epos and tutorial-style documentation are available from github at https://github.com/EvolBioInf/epos. The starting point of the estimation procedure assumes a constant population size throughout the entire history of the sample. The maximum-likelihood estimator of $N_{e}$ is then equivalent to Watterson’s (1975) estimator. The next most complicated model involves a single coalescent breakpoint *k* flanked by two different $N_{e}$, such that $N_{i}$ is a constant $N_{k}$ for k<i≤n, and $N_{k-1}$ for i≤k. The formulae for the expected SFS then reduce to a three-parameter model, whose solution requires a search for the combination of k, $N_{k}$, and $N_{k-1}$ that maximizes the composite likelihood of the observed SFS, which can be found by Newton-Raphson iteration. This procedure is then iterated in a stepwise fashion, with each iteration increasing the number of breakpoints by one, until the difference in adjacent likelihoods no longer improves beyond a critical value. To this end, we employ the Akaike Information Criterion (AIC), moving on to the next iteration provided the log-likelihood has increased by at least 2.0 in the preceding iteration. The end result is a stepwise plot of interval-specific $N_{e}$ estimates, with the breakpoints converted to time (in generations) using the interval-specific expected coalescent times given by Equation (2) and a mutation rate provided by the user.

Several additional features are built into epos. First, it is possible to analyze folded as well as unfolded SFSs. Second, the auxiliary program bootSfs (github.com/EvolBioInf/bootSfs) implements the bootstrap to estimate the sampling variance of the estimated demographic history. Third, it is possible to exclude classes from the SFS, if for example the singleton class is deemed unreliable owing to sequencing errors. Fourth, the user can specify the AIC stopping criterion. Fifth, all possible combinations of breakpoint locations can be re-evaluated at each iteration, as opposed to sequentially adding fixed breakpoints; to accomplish this, there are two versions of the function nextConfig in epos: a fast, greedy version, which adds one new level at a time and a slow, exhaustive version, which goes through all possible combinations of levels. This flexibility is provided because the the number of possible sets of breakpoints increases exponentially as the stepwise estimation procedure advances.

**Figure 2:**
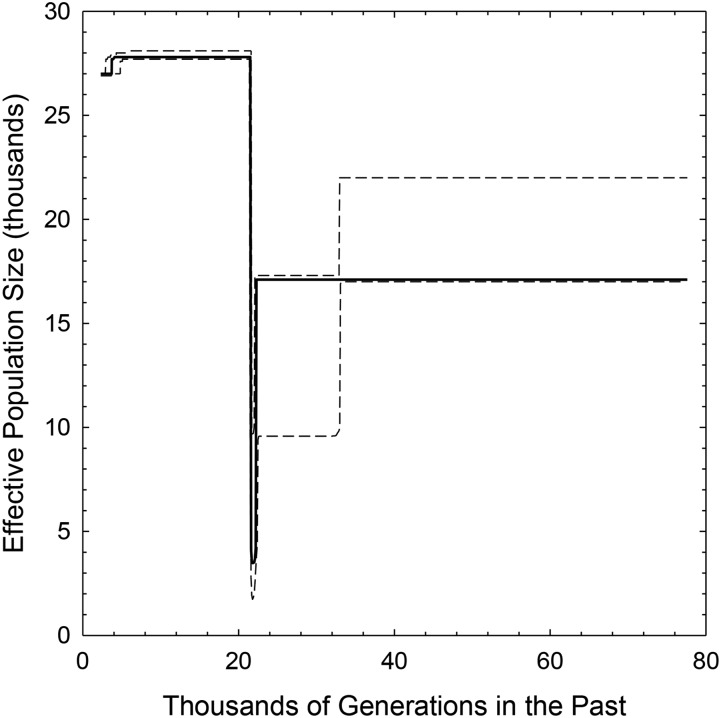
Results from the application of epos to the Yoruba SNP data set applied in Lapierre *et al.* (2017). The Yoruba data set was bootstrapped 10,000 times (average given by solid line) and the 5 and 95% quantiles (lower and upper dashed lines, respectively) computed from these replicates.

Based on the performance of the Stairway Plot algorithm of Liu and Fu (2015), as applied to a single human (Yoruba) population sample, Lapierre *et al.* (2017) have raised concerns about the use of model-flexible approaches to estimating historical demography, as opposed to using model-constrained approaches that pre-specify the form of population growth and breakpoints in demographic features. In this particular application, these authors showed that the Stairway Plot algorithm predicts a complex demographic history with multiple recent bottlenecks, with a poor least-squares fit to the observed SFS (with a weighted mean-squared distance of $d^{2}=2.9\times 10^{-3}$). In contrast, simpler pre-specified models (*e.g.*, linear, exponential, and sudden) predicted consistent increases in population size to the present (all with $d^{2}$ in the range of $2.2\times 10^{-4}$ to $4.1\times 10^{-4}$). Application of epos to the same data set predicts an increase in population size from the deep past to the present, but with a short intervening population bottleneck ∼500,000 years ago (Figure 2), and has a fivefold reduction of $d^{2}$ to $8.0\times 10^{-5}$. The current $N_{e}≃28,000$ predicted by epos is comparable to that obtained by other methods. Thus, contrary to the conclusions of Lapierre *et al.* (2017), these results suggest that constrained models are not inherently superior to flexible models, but simply that the quality of the results obtained in the latter context can be suboptimal if the algorithmic approach of Liu and Fu (2015) is applied.

**Figure 3:**
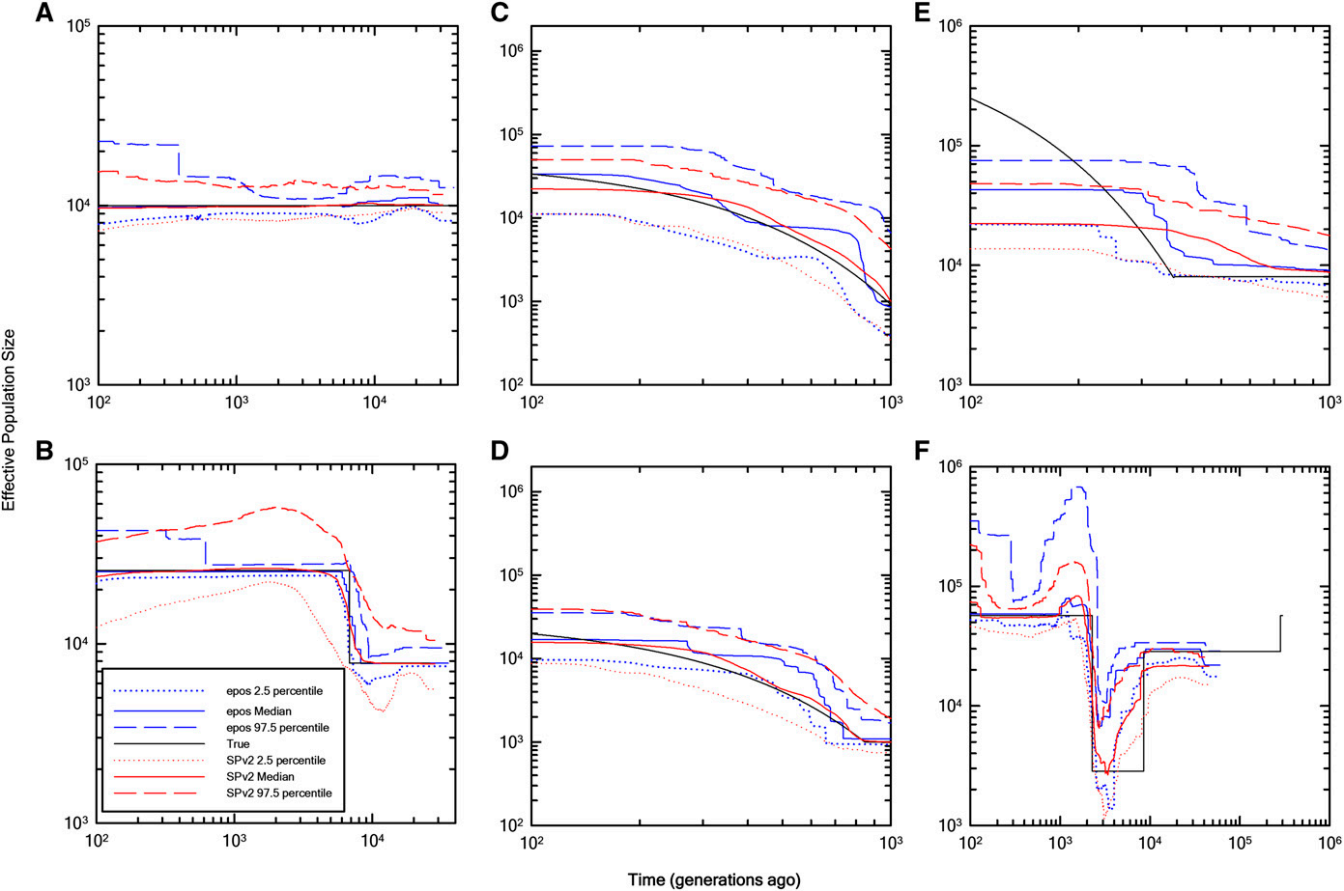
Comparison of the estimation quality of epos (blue) to results from the Stairway Plot (SP, red) method of Liu and Fu (2015). In each panel, the means of 10 medians and 5 and 95% quantiles are shown (dotted and dashed lines, based on averages of 10 independently derived SFSs). The black lines denote the true underlying demography used to simulate the data.

We have further evaluated the utility of epos by fitting $N_{e}$ histories to various demographic scenarios by generating sample SFSs using the coalescent software of Kelleher *et al.* (2016) and Chen *et al.* (2009) in the analyses in Figures 3A-E and 3F, respectively. Comparison of our results to those obtained by the algorithms of Liu and Fu (2015) shows that epos performs as well and in some cases better than the Stairway method (Figure 3). For each evaluated scenario, ten SFSs were generated, and 2,000 bootstrap replications were used to find the mean and percentiles of the effective-population size estimates, except under the last scenario (F), where 200 bootstrap replications were used. Epos is at least 1,000 times faster than Liu and Fu’s (2015) Stairway procedure. For example, epos and Stairway Plot v2 took 0:00:14 and 6:11:51, respectively, to analyze one site-frequency spectrum under the scenario in Figure 3a.

## Application to Daphnia population-genomic data

We applied epos to the SFSs from eight *Daphnia pulex* populations, an emerging model system in population genomics. A practical issue in any population-genomic study with moderate sequence coverage per site is that not all sites are scored in identical numbers of individuals. In this particular study, for each population 8 to 14 SFSs were available for sample sizes of 40,000 to 2,400,000 nucleotides (Table 1), with separate analyses performed for fourfold redundant silent sites in protein-coding genes and internal intron sites known to behave in a nearly neutral fashion (Lynch *et al.* 2017). Although the individuals used within these within-population analyses were largely overlapping, the sites employed were fully nonoverlapping. The numbers of individuals associated with each SFS range from 62 to 93.

**Table 1:** The site-frequency spectra analyzed in this study. Details on data acquisition can be found in Maruki *et al.* (2019) For each population, 8 to 14 SFSs were used, with a range of numbers of sampled individuals and nucleotides as noted in the text

Population	Number of SFSs	Sample Sizes	Nucleotides
CHQ	8	90 to 93	148,485 to 2,386,879
KAP	14	72 to 78	111,372 to 893,885
LPA	8	83 to 86	46,126 to 449,970
LPB	10	80 to 84	122,659 to 984,400
NFL	8	86 to 89	135,090 to 1,933,077
PA	12	62 to 67	63,013 to 101,901
POV	8	68 to 71	62,784 to 2,344,877
TEX	12	66 to 71	204,372 to 480,031

**Figure 4:**
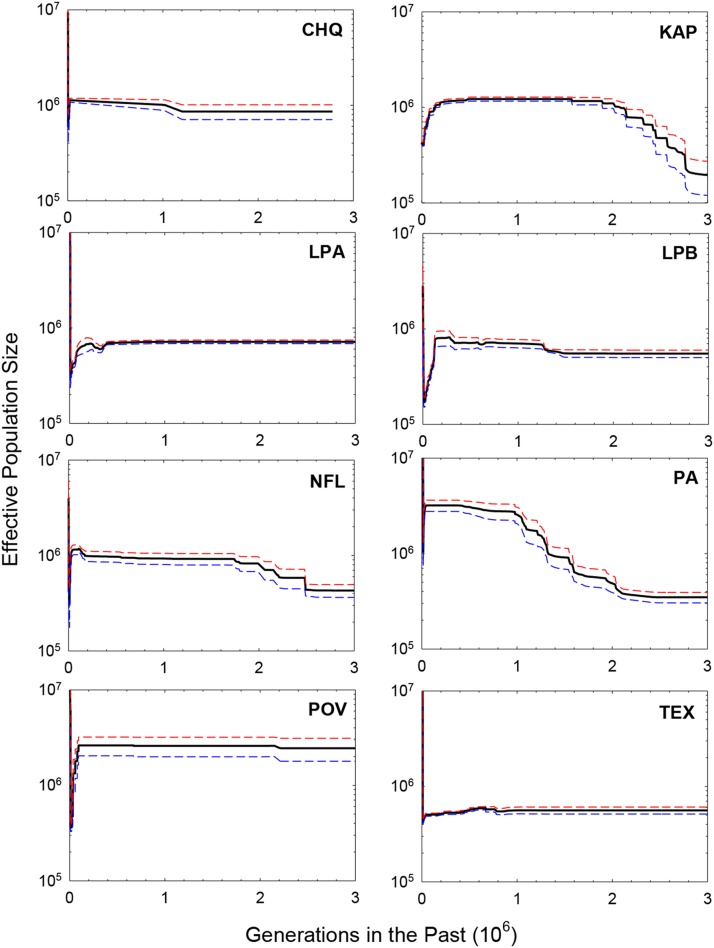
Estimated demographic history of eight *Daphnia pulex* populations, measured as the means of the medians (thick black lines) of the replicated 10,000 bootstrap estimates derived for the number of samples noted in Table 1. Deviations of single standard errors of the means are given as red and blue dashed lines.

**Figure 5:**
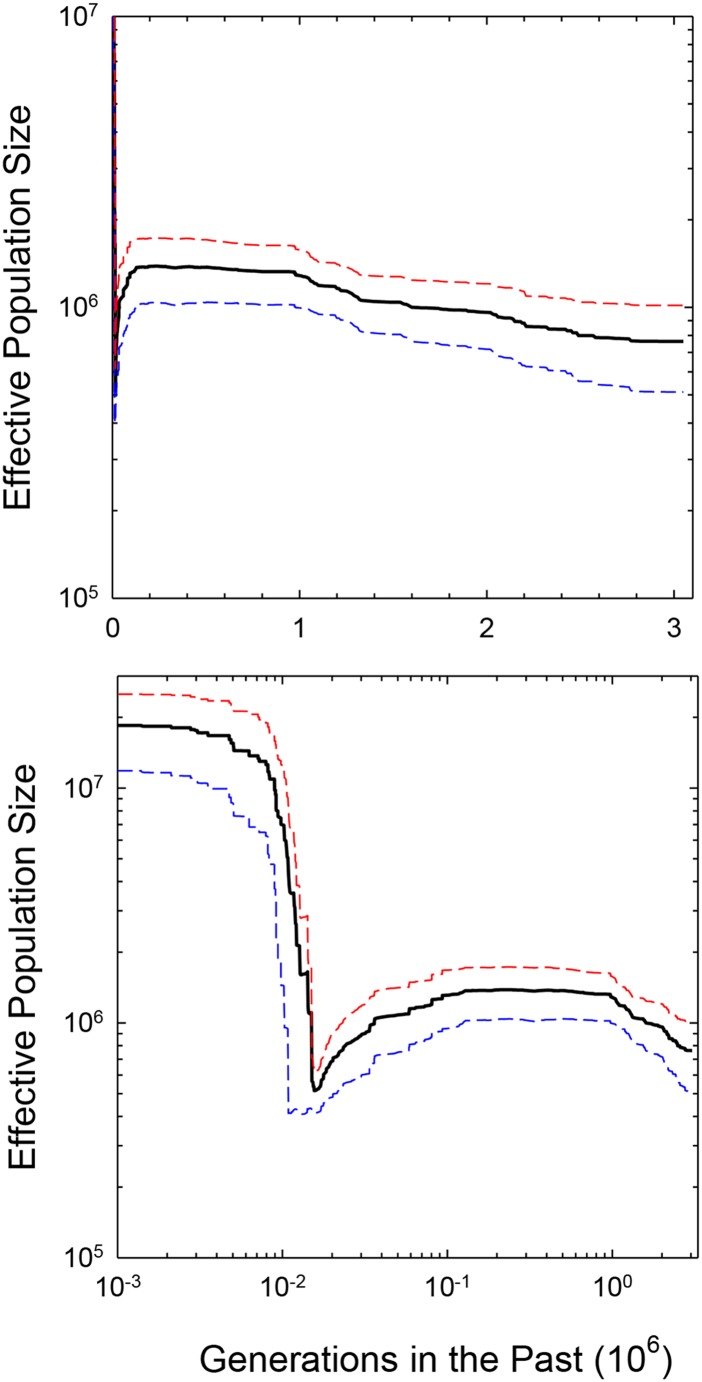
Average pattern of demographic history over the entire *D. pulex* metapopulation, plotted on the linear and logarithmic time scale, with deviations from the mean given as single standard errors among the profiles for each of the eight populations.

This type of partitioning is required because the SFS theory involves discrete distributions, *i.e.*, frequencies from different sample sizes should not be amalgamated into a single pooled SFS. However, such replication in analysis also provides some guard against sampling variance issues. For each of the samples, 10,000 bootstraps of the SFS were performed to generate a median demographic-history estimate, assuming a mutation rate of $u=5.7\times 10^{-9}$ per site per generation (Keith *et al.* 2016). The final demographic-history estimates for each population are then given as the means of separate median estimates (Figure 4), and a further summary mean over all populations is given in Figure 5.

Although there are significant differences among populations, these analyses suggest a fairly consistent demographic history among all populations (focusing on an order-of-magnitude time scale). From $∼10^{5}$ to $3\times 10^{6}$ generations in the past, $N_{e}$ was almost always in the range of 0.5 to $2.0\times 10^{6}$, with little evidence of dramatic changes. All populations exhibit evidence of a twofold or so decline in $N_{e}$ in the very recent past, followed by an interval of population-size expansion around 20,000 generations ago (Figure 5). Assuming five to ten generations per year, these recent demographic shifts would represent post-Pleistocene changes, with the point of initiation of population-size expansion being 2,000 to 4,000 years ago (roughly corresponding to the Neopluvial, a period of wetter and cooler climate in North America). The ending points in the demographic profiles ($∼3\times 10^{6}$ million years ago) fall in the mid-Pleistocene. Influences from European settlement, deforestation, and agriculture would date no further back than 5,000 generations, and are not discernible.

**Figure 6:**
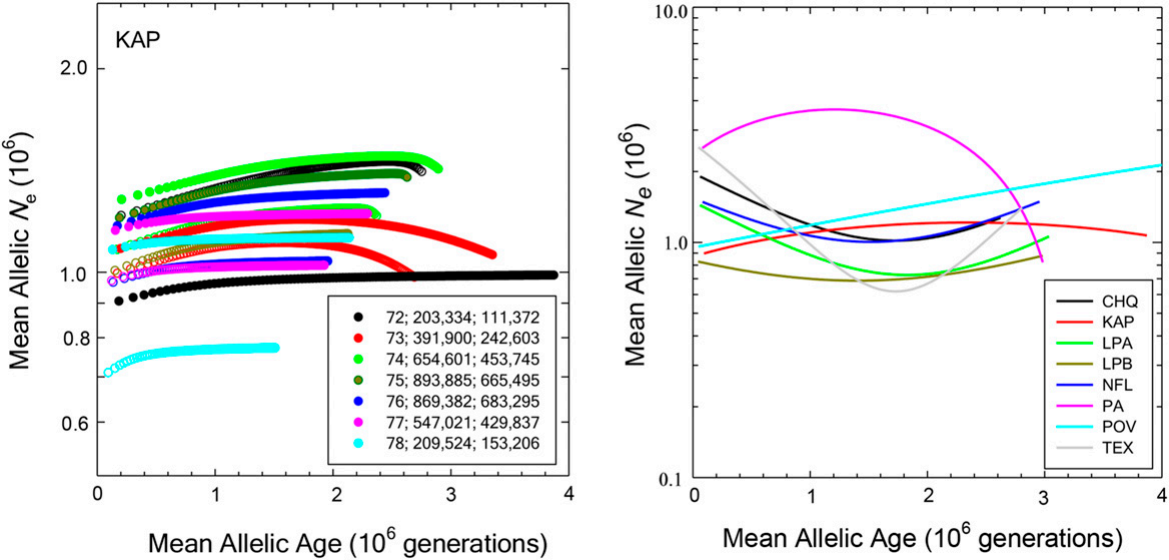
Left) The average effective population sizes and ages associated with each site-frequency class of alleles for the KAP population of *D. pulex*, with open symbols for internal intron sites (as defined in Lynch *et al.* 2017) and closed symbols for fourfold redundant sites. Sample sizes ranging from 72 to 78 diploid individuals have associated numbers of scored sites given in the inset for internal intron sites and fourfold redundant sites, respectively. Right) Summary of the results for each population, simply given as best second-order polynomial fits to the full sets of data.

Finally, the relationships between the mean $N_{e}$ of SNPs and their average age is given for each population in Figure 6. The left panel provides an example of the variation among sample-size classes for one particular population (with each point representing a particular SFS class for a particular number of individuals scored). The right panel summarizes the average results for each population as simple first- or second-order polynomial least-squares regressions. The main point again is that these *Daphnia* populations do not exhibit major demographic shifts across allele-frequency classes, with the population average $N_{e}$ associated with SNPs of all ages almost always falling in the range of 800,000 to 3,000,000.

### Data availability

The details on data acquisition, curation, and deposition appear in Maruki and Lynch (2019); the FASTQ files of the raw-sequence data are publicly available via the NCBI Sequence Read Archive (accession number SRP155055). Supplemental material available at figshare: https://doi.org/10.25387/g3.10265867.

## Discussion

The methods developed herein provide a model-independent means for estimating the past demographic history of a sample, using information on the frequency distribution of nucleotide sites assumed to behave in a neutral to nearly neutral manner. The approach taken assumes that changes in population size occur only at specific points in a genealogy, *i.e.*, at the times of average occurrence of coalesence events. This, of course, will never happen precisely in any natural population. However, as the times of coalescent events vary widely among genealogies, such granularity can be expected to average out. Moreover, the approach taken does provide an increasingly fine dissection of the overall time scale under evaluation with increasing sample size (*n*). So as shown by Liu and Fu (2015) and herein, the method has the potential to closely approximate the more continuous patterns of population-size changes that likely occur in nature.

In the proposed method of estimation, Epos simply starts with an assumption of constant $N_{e}$, and then progressively searches for points of change in $N_{e}$ that, when invoked, yield significant improvements in the likelihood of the observed SFS data in a stepwise manner. Application of the bootstrap yields a further smoothing of the output estimates as well as confidence intervals on the overall pattern. Further smoothing is obtained by partitioning the SFS data into classes differing in sample sizes (or from different classes of sites, such as fourfold redundant codon sites *vs.* internal intron sites, both of which behave in a nearly neutral fashion). SFS sample-size variation will generally be the rule in low-coverage population-genomic sequencing data, where some individuals will have inadequate sequence at random sites.

The theoretical basis of the methods described herein is the same as that adopted by Liu and Fu (2015), although we have derived a number of extensions. In addition, the estimation procedures embodied in Epos deliver advances over the pioneering work of Liu and Fu (2015) in a number of ways. First, by using a Newton method for maximizing the likelihood rather than a (slow) genetic algorithm for optimization, the overall algorithmic approach is considerably more efficient, improving computational speed by over an order of magnitude without sacrificing accuracy in estimation (and in some cases apparently improving it). Second, Epos is capable of an exhaustive search for the best-fit demographic scenario, up to a number of steps specified by the user. Under this exhaustive search model, in adding a new breakpoint to the demography, each step in the iterative fitting re-evaluates the positions of all preceding breakpoints and their flanking $N_{e}$ estimates. Third, epos returns estimates on the average ages and $N_{e}$ (and sampling errors) of alleles within different frequency classes.

One potential concern with our method is its reliance on a composite-likelihood approach that ignores the nonindependence of linked SNPs. There are two reasons to believe that this is a minor issue with respect to the final analyses. First, most organisms have ten or more chromosomes, so only a minor fraction of pairs of loci are even on the same chromosome, and even a smaller fraction are within the $∼10^{6}$ bp where linkage disequilibrium is likely to be significant. Second, our simulation studies on algorithm performance, which generated data based on a recombining chromosome and then applied the composite likelihood, did indeed yield results consistent with simulated demographies. Although desirable, full-likelihood methods allowing for linked loci would be enormously computationally demanding, but more importantly would require detailed information on chromosomal map structures, which are available for few species.

Like all polymorphism-based methods, our approach is expected to become increasingly unreliable at very distant times in the past, owing to the increasing granularity of the coalescent process, and the fact that few polymorphisms are expected to survive for more than $4N_{e}$ generations. In addition, the ability to estimate very recent population-size changes is a function of the sample size, as there can be no power to estimate a span of time during which there is essentially zero chance of a *de novo* mutation appearing in a sample.

## APPENDIX

Results at various intermediate steps had to be derived before arriving at the main results of the text, some scattered in the prior literature (*e.g.*, Janson and Kersting 2010; Dahmer and Kersting 2015). Here we summarize things in one place to make the overall approach more transparent.

### Probability distribution for external branches.

By definition, singleton mutations exist only on external branches, which always start at level k=n in the coalescent and can end at levels k=n−1 to 1, where *n* is the sample size. These locations denote the consecutive coalescence events across the tree (with k=1 denoting the base of the genealogy). We wish to determine the probability distribution of branch lengths in units of coalescence events across the tree, with $P_{n,k}\left(1\right)$ denoting the probability that an external branch (starting at level *n*) ends at level *k*. This can be accomplished by letting $S_{n,k}$ denote the probability that a singleton branch descends to at least level *k* without having been absorbed by a coalescence event. By definition, $S_{n,n}=1$, and

$$P_{n,k}\left(1\right)=S_{n,k+1}-S_{n,k}.$$ (A1)

$S_{n,k}$ is the fraction of singleton branches extending down the tree until at least level k, whereas $P_{n,k}\left(1\right)$ denotes the fraction of all singleton branches terminating at level k.

To obtain the probability of a particular external branch surviving until the first coalescence event across the tree, note that two draws must be made without replacement from the *n* initial branch tips. The probability that neither draw involves the focal branch tip is

$$S_{n,n-1}=\left(1-\frac{1}{n}\right)\left(1-\frac{1}{n-1}\right)=\frac{n-2}{n}.$$ (A2a)

The probability of the focal branch tip continuing to survive the next coalescence event (*i.e.*, not being drawn) is obtained by noting that there are now n−1 possible draws,

$$S_{n,n-2}=S_{n,n-1}\cdot \left(1-\frac{1}{n-1}\right)\left(1-\frac{1}{n-2}\right)=\frac{\left(n-2\right)\left(n-3\right)}{n\left(n-1\right)},$$ (A2b)

which generalizes to,

$$S_{n,n-x}=\frac{\left(n-x\right)\left(n-x-1\right)}{n\left(n-1\right)}.$$ (A3)

Substituting into Equation (A1) yields the general expression for a singleton branch terminating at the *x*th coalescence event in the tree,

$$P_{n,n-x}\left(1\right)=\frac{2\left(n-x\right)}{n\left(n-1\right)}.$$ (A4)

### Probability distribution for internal branches

The situation is more complicated with internal branches, which have variable starting and ending points, and also vary in number among alternative tree topologies. However, progress is made possible with a result from Dahmer and Kersting (2015), which states that for a sample of size *n* the expected number of segments of order *r* present at level *k* (just below the coalescence at this point) is

$$W_{k}\left(r\right)=\frac{k\left(k-1\right)}{n-r}\prod_{i=0}^{r-2}\left[\frac{n-k-i}{n-1-i}\right]=\left(\frac{k\left(k-1\right)}{n-r}\right)\cdot \frac{\left(n-k\right)!\left(n-r\right)!}{\left(n-k-r+1\right)!\left(n-1\right)!},$$ (A5)

for k≤(n−r+1), and $W_{k}\left(r\right)=0$ for k>(n−r+1). This result can also be obtained by extrapolating Equation (14) in Fu (1995). Again note that k=n denotes the branch-tip level, k=n−1 denotes the first coalescence event in the sample, and k=1 denotes the base of the genealogy (the final coalescent), so that starting points (nearer the branch tips) have higher integer values than ending points.

From this expression, it is possible to deconvolute the expected number of internal branches initiating at level k, $B_{k}\left(r\right)$, by noting the birth-death process involving segments of order *r* as one descends down the tree,

$$W_{k}\left(r\right)=\left[W_{k+1}\left(r\right)\cdot s_{k}\right]+B_{k}\left(r\right),$$ (A6a)

where

$$s_{k}=\left(1-\frac{1}{k+1}\right)\left(1-\frac{1}{k}\right)=\frac{k-1}{k+1},$$ (A6b)

is the probability that a particular group at level k+1 is not a participant in the next coalescence event. Upon rearrangement, and substitution from above,

$$B_{k}\left(r\right)=\frac{r-1}{n-k}\cdot W_{k}\left(r\right).$$ (A7)

The highest level for a nonzero value of this birth rate is n−r+1, and thereafter $0<B_{k}\left(r\right)<1$, except for the case of k=n−1 for r=2, $B_{n-1}\left(2\right)=1,$ as the first coalescence event always produces a doubleton.

From the statistical properties of coalescence events arising subsequent to the origin of a segment noted above, the expected number of internal branches of order *r* initiating at level *k* and ending at level k−x, where 1≤x≤(k−1), is

$$\begin{array}{l} B_{k,k-x}\left(r\right)=B_{k}\left(r\right)\cdot \frac{2}{k-x+1}\cdot \prod_{i=1}^{x-1}\left[1-\frac{2}{k-i+1}\right]\\ =\frac{2\left(k-x\right)B_{k}\left(r\right)}{k\left(k-1\right)}, \end{array}$$ (A8)

which follows directly from Equation (A4).

The complete probability distribution for segment spans of order *r* is then

$$P_{k,k-x}\left(r\right)=\frac{B_{k,k-x}\left(r\right)}{B_{T}\left(r\right)},$$ (A9a)

where the denominator is the expected total number of branches of order *r* in a genealogy

$$B_{T}\left(r\right)=\sum_{k=2}^{n-r+1}B_{k}\left(r\right).$$ (A9b)

None of the features in this entire section on the genealogical structure of a sample depend on demographic history, although the lengths of the individual branches do.

### Average age of a SNP

Griffiths (2003) obtained a general expression for the average age of a derived allele of arbitrary frequency under the assumption of constant population size, given in generations,

$$E\left[A_{r}\right]=\frac{4N_{e}r}{n-r}\sum_{x=r+1}^{n}\frac{1}{x}.$$ (A10)

However, here we are concerned with the more complex issue of estimating the average age of SNPs when the population size is not constant. The central challenge is that mutations of various orders can appear on branches that start and end at various levels in the tree, each of which may be associated with a particular $N_{e}.$

Here, we take advantage of a derivation of Griffiths and Taveré (1998), their Equation (5.4), which requires a definition of $p_{n,k}\left(r\right)$, the probability that a random line, at the time there are *k* total lines in the coalescent, is subtended by *r* leaves in the tree. This is equivalent to $W_{k}\left(r\right)/k,$ with simplification of Equation (A5) leading to

$$p_{n,k}\left(r\right)=\frac{k-1}{r}\cdot \frac{\left(\begin{array}{c} n-k\\ r-1 \end{array}\right)}{\left(\begin{array}{c} n-1\\ r \end{array}\right)}.$$ (A11)

Letting $t_{k}$ be the time the coalescent has *k* lines (defined by Equation (2) in the main text), and $T_{k+1}=t_{n}+\cdots +t_{k+1}$, then as Griffiths and Tavare (1998) argue above their Equation (5.1), the expected age of an allele arising on a branch when the coalescent has *k* lines, is $Ut_{k}+T_{k+1}$, where U is a uniformly distributed on [0,1] independent of all other random variables.

To obtain the moments of the ages, we take advantage of a derivation of Griffiths and Taveré (1998), their Equation (5.4),

$$E[A_{r}^{j}]=\frac{\frac{1}{j+1}\sum_{k=2}^{n}\left(\begin{array}{c} n-k\\ r-1 \end{array}\right)k\left(k-1\right)E\left[T_{k}^{j+1}-T_{k+1}^{j+1}\right]}{\sum_{k=2}^{n}\left(\begin{array}{c} n-k\\ r-1 \end{array}\right)k\left(k-1\right)E\left[t_{k}\right]}.$$ (A12)

The denominator, which is independent of *j*, reduces to

$$\sum_{k=2}^{n}\left(\begin{array}{c} n-k\\ r-1 \end{array}\right)k\left(k-1\right)E\left[t_{k}\right]=4\sum_{k=2}^{n}\left(\begin{array}{c} n-k\\ r-1 \end{array}\right)N_{k}.$$ (A13)

For the numerator, we just provide the results necessary for the first two moments, which are required for estimates of the mean and variance of the average age. For j=1

$$E\left[T_{k}^{2}-T_{k+1}^{2}\right]=E\left[t_{k}^{2}+2t_{k}\sum_{\ell =k+1}^{n}t_{\ell }\right]=2\frac{{\left(2N_{k}\right)}^{2}}{{\left(\begin{array}{c} k\\ 2 \end{array}\right)}^{2}}+2\frac{2N_{k}}{\left(\begin{array}{c} k\\ 2 \end{array}\right)}\sum_{\ell =k+1}^{n}\frac{2N_{\ell }}{\left(\begin{array}{c} \ell \\ 2 \end{array}\right)}=2\frac{2N_{k}}{\left(\begin{array}{c} k\\ 2 \end{array}\right)}\sum_{\ell =k}^{n}\frac{2N_{\ell }}{\left(\begin{array}{c} \ell \\ 2 \end{array}\right)},$$

which leads to

$$\frac{1}{2}\sum_{k=2}^{n}\left(\begin{array}{c} n-k\\ r-1 \end{array}\right)k\left(k-1\right)E\left[T_{k}^{2}-T_{k+1}^{2}\right]=16\sum_{k=2}^{n}N_{k}\left(\begin{array}{c} n-k\\ r-1 \end{array}\right)\sum_{\ell =k}^{n}\frac{N_{\ell }}{\ell \left(\ell -1\right)}.$$ (A14)

For j=2,

$$\begin{array}{c} E\left[{\left(\sum_{\ell =k+1}^{n}t_{\ell }\right)}^{2}\right]=\sum_{\ell =k+1}^{n}E\left[t_{\ell }^{2}\right]+2\sum_{\ell =k+1}^{n}\sum_{m=\ell +1}^{n}E\left[t_{\ell }t_{m}\right]=2\sum_{\ell =k+1}^{n}\frac{{\left(2N_{\ell }\right)}^{2}}{{\left(\begin{array}{c} \ell \\ 2 \end{array}\right)}^{2}}+2\sum_{\ell =k+1}^{n}\sum_{m=\ell +1}^{n}\frac{2N_{\ell }}{\left(\begin{array}{c} \ell \\ 2 \end{array}\right)}\frac{2N_{m}}{\left(\begin{array}{c} m\\ 2 \end{array}\right)}\\ =2\sum_{\ell =k+1}^{n}\sum_{m=\ell }^{n}\frac{2N_{\ell }}{\left(\begin{array}{c} \ell \\ 2 \end{array}\right)}\frac{2N_{m}}{\left(\begin{array}{c} m\\ 2 \end{array}\right)} \end{array}$$

and therefore

$$\begin{array}{l} E\left[T_{k}^{3}-T_{k+1}^{3}\right]=E\left[t_{k}^{3}+3t_{k}{\left(\sum_{\ell =k+1}^{n}t_{\ell }\right)}^{2}+3t_{k}^{2}\sum_{\ell =k+1}^{n}t_{\ell }\right]\\ =6\frac{{\left(2N_{k}\right)}^{3}}{{\left(\begin{array}{c} k\\ 2 \end{array}\right)}^{3}}+6\frac{2N_{k}}{\left(\begin{array}{c} k\\ 2 \end{array}\right)}\sum_{\ell =k+1}^{n}\sum_{m=\ell }^{n}\frac{2N_{\ell }}{\left(\begin{array}{c} \ell \\ 2 \end{array}\right)}\frac{2N_{m}}{\left(\begin{array}{c} m\\ 2 \end{array}\right)}+6\frac{{\left(2N_{k}\right)}^{2}}{\left(\begin{array}{c} k\\ 2 \end{array}\right)}\sum_{\ell =k+1}^{n}\frac{2N_{\ell }}{\left(\begin{array}{c} \ell \\ 2 \end{array}\right)}\\ =6\frac{2N_{k}}{\left(\begin{array}{c} k\\ 2 \end{array}\right)}\sum_{\ell =k}^{n}\sum_{m=\ell }^{n}\frac{2N_{\ell }}{\left(\begin{array}{c} \ell \\ 2 \end{array}\right)}\frac{2N_{m}}{\left(\begin{array}{c} m\\ 2 \end{array}\right)}, \end{array}$$

which leads to

$$\frac{1}{3}\sum_{k=2}^{n}\left(\begin{array}{c} n-k\\ r-1 \end{array}\right)k\left(k-1\right)E\left[t_{k}^{3}-t_{k+1}^{3}\right]=128\sum_{k=2}^{n}N_{k}\left(\begin{array}{c} n-k\\ r-1 \end{array}\right)\sum_{\ell =k}^{n}\frac{N_{\ell }}{\ell \left(\ell -1\right)}\sum_{m=\ell }^{n}\frac{N_{m}}{m\left(\ell -1\right)}.$$ (A15)

Substitution of Equations (A13-15) into Equation (A12) leads to the expressions for the mean and variance in the main text, Equations (6a,b).

### Average $N_{e}$ of a SNP

The results from the previous section summarize the average amounts of times that alleles spend at the various population sizes. For SNPs of any order *r*, the average $N_{e}$ experienced can be determined by weighting the relative time spent in each interval by the interval-specific population sizes. From Griffiths and Taveré (1998), their Equation (5.1), we know that (6a) is in fact equivalent to

$$E\left[A_{r}\right]=\frac{\sum_{k=2}^{n}kp_{n,k}\left(r\right)E\left[t_{k}\cdot \left(Ut_{k}+T_{k+1}\right)\right]}{\sum_{k=2}^{n}kp_{n,k}\left(r\right)E\left[t_{k}\right]},$$

where *U* is uniformly distributed on [0,1]; see also the explanation below (A11). This formula can be interpreted such that the $kp_{n,k}$-term is proportional to the probability that the SNP of size *r* occurs within level *k*. The expectation in the numerator then gives the time how long in the past this level happened. For estimating the average $N_{e}$ of a SNPs in size *r*, we need to weigh this expectation by the experienced population sizes, leading us to

$$E\left[P_{r}\right]=\frac{\sum_{k=2}^{n}kp_{n,k}\left(r\right)E\left[t_{k}\cdot \left(Ut_{k}N_{k}+t_{k+1}N_{k+1}+\cdots +t_{n}N_{n}\right)\right]}{\sum_{k=2}^{n}kp_{n,k}\left(r\right)E\left[t_{k}\cdot \left(Ut_{k}+T_{k+1}\right)\right]}.$$

For the denominator, note that

$$E\left[U\right]=\frac{1}{2},\;E\left[t_{k}^{2}\right]=\frac{8N_{k}^{2}}{{\left(\begin{array}{c} k\\ 2 \end{array}\right)}^{2}}=\frac{32N_{k}^{2}}{k^{2}{\left(k-1\right)}^{2}},\;E\left[T_{k+1}\right]=\sum_{\ell =k+1}^{n}\frac{4N_{\ell }}{\ell \left(\ell -1\right)}.$$

So, we compute

$$\begin{array}{l} \sum_{k=2}^{n}kp_{n,k}\left(r\right)E\left[t_{k}\cdot \left(Ut_{k}+t_{k+1}+\cdots +t_{n}\right)\right]\\ =\sum_{k=2}^{n}\frac{\left(\begin{array}{c} n-k\\ r-1 \end{array}\right)k\left(k-1\right)}{\left(\begin{array}{c} n-1\\ r \end{array}\right)r}\left(\frac{16N_{k}^{2}}{k^{2}{\left(k-1\right)}^{2}}+\frac{4N_{k}}{k\left(k-1\right)}\overset{n}{\underset{\ell =k+1}{\sum }}\frac{4N_{\ell }}{\ell \left(\ell -1\right)}\right)=\sum_{k=2}^{n}\frac{4N_{k}\left(\begin{array}{c} n-k\\ r-1 \end{array}\right)}{\left(\begin{array}{c} n-1\\ r \end{array}\right)r}\sum_{\ell =k}^{n}\frac{4N_{\ell }}{\ell \left(\ell -1\right)}. \end{array}$$

The numerator is actually almost the same except for a different weight of the $N_{\ell }$’s, *i.e.*

$$\sum_{k=2}^{n}kp_{n,k}\left(r\right)E\left[t_{k}\cdot \left(Ut_{k}N_{k}+t_{k+1}N_{k+1}+\cdots +t_{n}N_{n}\right)\right]=\sum_{k=2}^{n}\frac{4N_{k}\left(\begin{array}{c} n-k\\ r-1 \end{array}\right)}{\left(\begin{array}{c} n-1\\ r \end{array}\right)r}\sum_{\ell =k}^{n}\frac{4N_{\ell }^{2}}{\ell \left(\ell -1\right)}.$$

Dividing the last two displays then gives Equation (8).
